# Network Allocation Vector (NAV) Optimization for Underwater Handshaking-Based Protocols

**DOI:** 10.3390/s17010032

**Published:** 2016-12-24

**Authors:** Junho Cho, Ethungshan Shitiri, Ho-Shin Cho

**Affiliations:** School of Electronics Engineering, Kyungpook National University, Daegu 41566, Korea; jh_cho@ee.knu.ac.kr (J.H.); ethungshan@ee.knu.ac.kr (E.S.)

**Keywords:** CSMA/CA, hidden node, MAC, NAV, throughput, underwater acoustic sensor networks

## Abstract

In this paper, we obtained the optimized network allocation vector (NAV) for underwater handshaking-based protocols, as inefficient determination of the NAV leads to unnecessarily long silent periods. We propose a scheme which determines the NAV by taking into account all possible propagation delays: propagation delay between a source and a destination; propagation delay between a source and the neighbors; and propagation delay between a destination and the neighbors. Such an approach effectively allows the NAV to be determined precisely equal to duration of a busy channel, and the silent period can be set commensurate to that duration. This allows for improvements in the performance of handshaking-based protocols, such as the carrier sense multiple access/collision avoidance (CSMA/CA) protocol, in terms of throughput and fairness. To evaluate the performance of the proposed scheme, performance comparisons were carried out through simulations with prior NAV setting methods. The simulation results show that the proposed scheme outperforms the other schemes in terms of throughput and fairness.

## 1. Introduction

Underwater communication has been receiving tremendous attention due to its application in underwater areas where it is difficult for humans to be constantly present. Such applications include marine resource exploration, pollution monitoring, surveillance, and oceanic environment observations. The unique characteristic of underwater channel limits the use of radio frequency (RF) waves in underwater networks. That is because RF waves experience extensive attenuation and fading losses in liquid media. Therefore, acoustic waves have been the preferred form of communication in underwater networks, as the acoustic waves are less affected by the aforementioned losses and can be used over long distances [1]. Generally, the networks that use acoustic waves are called underwater acoustic sensor networks (UWASNs). Nevertheless, acoustic waves are limited in operational bandwidth, suffer from a higher bit error rate (BER), and introduce a relatively large propagation delay compared to RF waves of terrestrial sensor networks [2].

To achieve higher throughputs in UWASNs, the requirement of an efficient underwater medium access control (MAC) protocol that can handle large propagation delays cannot be ignored [3,4]. Contention-based MAC protocols are highly preferred for UWASNs as they have the advantage of utilizing the full bandwidth of the underwater channel [5]. They are classified into random-access protocols and handshaking protocols. Several underwater random-access protocols have been proposed such as the slotted ALOHA (S-ALOHA), ALOHA with carrier sense (ALOHA-CS), underwater wireless acoustic networks MAC (UWAN-MAC), and tone-Lohi (T-Lohi) [6,7,8,9]. While these protocols are simple, collisions are still inevitable when multiple nodes try to access the channel simultaneously as they do not explicitly reserve the channel. On the other hand, handshaking protocols reserve the channel by exchanging several control packets between nodes, thereby avoiding collisions. This makes handshaking protocols more suitable for UWASNs, and is evident from the large amount literature available—MACA for underwater (MACA-U), propagation-delay-tolerant collision avoidance protocol (PCAP), APCAP, receiver-initiated packet train (RIPT), delay-aware opportunistic transmission scheduling (DOTS), CS-MAC, reservation-based MAC (R-MAC), ROPA, Spatially fair MAC (SF-MAC), floor acquisition multiple access (FAMA), contention-based parallel reservation MAC (COPE-MAC), cascading multi-hop reservation and transmission (CMRT), hybrid sender- and receiver-initiated protocol (HSR), dynamic NAV (DYNAV) and carrier sense multiple access/collision avoidance for underwater communications (UW-CSMA/CA) [10,11,12,13,14,15,16,17,18,19,20,21,22,23,24,25].

To avoid collisions, handshaking protocols rely on a system parameter called the network allocation vector (NAV). The NAV contains information on the duration for which the channel will be busy and allows a neighboring node to stay silent for that duration. If a neighboring node does not stay silent for that particular duration, then collisions may occur resulting from what is commonly known as the hidden-node problem. In terrestrial RF networks, the propagation delay is infinitesimally small and therefore the NAV duration is precisely equal to the communication time between a source and a destination [26]. However, in UWASNs where the propagation delay is large, the NAV duration must also include the propagation delay or else ignoring the propagation delay can result in an inaccurately small NAV duration. This may allow the hidden nodes to cause collisions as the hidden node will wake up before the existing communication is completed. A couple of studies have proposed a modified NAV that considers the propagation delays in underwater acoustic channels [24,25].

In [25], the authors proposed UW-CSMA/CA (CSMA/CA for underwater communication) in which an NAV contains the maximum propagation delay of a network, thereby avoiding collisions. However, the use of maximum propagation delay, regardless of the internodal distances, causes some degree of inefficiency in terms of resource utilization. A similar method was also used in the dynamic NAV (DYNAV) determination protocol [24]. Unlike UW-CSMA/CA, DYNAV uses the maximum propagation delay only once for the NAV associated with the request-to-send (RTS) packet, which is the first packet in the handshaking process. For the remaining handshaking, an estimated value of the propagation delay is used to determine the NAV to avoid any unnecessarily long NAVs. However, since DYNAV considers only the propagation delay between a source and a destination, and not between a source and the neighbors or between a destination and the neighbors, all neighbors have the same NAV, regardless of their distances from the source or destination. This means that a neighbor who is far from the source or destination would wake up much later than a closer neighbor, as the distant neighbor overhears the control packets and sets the NAV at a delayed time. Accordingly, the likelihood of a distant neighbor occupying the channel is very small which causes severe unfairness problems in the network.

In this paper, we propose a scheme to determine the NAV by taking into account the propagation delay of the underwater acoustic channel. However, unlike prior works, we take into account all possible propagation delays—propagation delay between a source and a destination; propagation delay between a source and the neighbors; and propagation delay between a destination and the neighbors. Such approach effectively determines an optimized NAV and eliminates any unnecessarily long silent periods. The NAV is constantly updated every time a new estimate of the propagation delay is obtained through the transmission time, which is time-stamped on to the overhearing handshaking packets. We show, through numerical experiments, that setting appropriate NAV or silent periods results in better throughput and fairness performance.

The rest of this paper is organized as follows. Section 2 discusses the problem statement. In Section 3, the proposed scheme is presented. The simulation results and discussions of the proposed scheme are presented in Section 4. Finally, in Section 5, we give the conclusions.

## 2. Problem Statement

**Figure 1:**
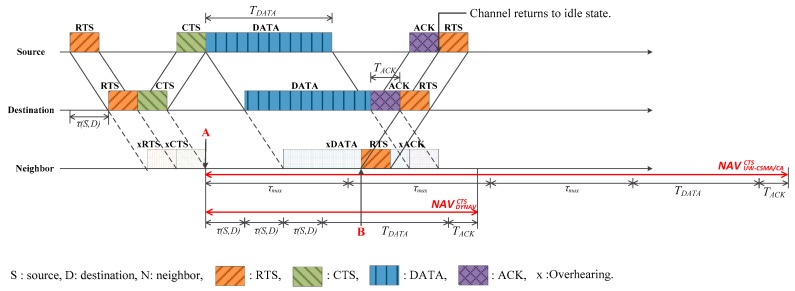
Example of network allocation vector (NAV) determination in conventional schemes and a highlight on the possibility of an early wake-up for a neighbor (denoted by point B). RTS: request-to-send; CTS: clear-to-send; DATA: data; ACK: acknowledgement; $\tau_{max}$: maximum propagation delay; τ(S,D) : source–destination propagation delay; DYNAV: dynamic NAV; UW-CSMA/CA: carrier sense multiple access/collision avoidance for underwater communications.

Figure 1 shows the NAV determination along with the handshaking procedure in a typical carrier sense multiple access/collision avoidance (CSMA/CA) protocol such as in UW-CSMA/CA and DYNAV. τ(i,j) and $\tau_{max}$ denote the propagation delay between nodes *i* and *j*, and the maximum propagation delay in the network, respectively. The handshaking takes place by exchanging packets in an orderly manner—RTS, clear-to-send (CTS), data (DATA), and acknowledgement (ACK). On overhearing the control packets such as RTS or CTS, the neighbors establish the NAV based on the transmission timing information contained in the packets, and later revise the NAV on overhearing a new control packet. The propagation delay between the source and destination is required to determine the NAV. However, because the exact propagation delay between the source and destination is unknown, the neighbors should estimate the value. In case of UW-CSMA/CA, the maximum propagation delay $\tau_{max}$ is used as shown in the Figure 1, where the neighbor sets the NAV on overhearing the CTS (point A) as:

(1) $$NAV_{UW-CSMA/CA}^{CTS}=3\tau_{max}+T_{DATA}+T_{ACK}$$

where $NAV_{Y}^{X}$ denotes the NAV calculated by overhearing the packet X of scheme Y, $3\tau_{max}$ corresponds to the sum of the three-way propagation delays associated with the CTS, data, and ACK, and $T_{X}$ denotes the transmission time of packet X. $\tau_{max}$ is the maximum propagation delay that can exist between two nodes in the network. It is shown that the NAV is unnecessarily long, which tends to degrade the channel utilization and throughput. In an effort to reduce the NAV, DYNAV [24] replaces $\tau_{max}$ with the source–destination propagation delay τ(S,D). Thus,

(2) $$NAV_{DYNAV}^{CTS}=3\tau (S,D)+T_{DATA}+T_{ACK}$$

The use of τ(S,D) definitely reduces NAV. However, considering the long propagation delay in the acoustic channel, the NAV is still too large compared to the communication time and should be reduced further. Let us consider a time instant, denoted by point B in Figure 1, such that the neighbor may transmit a new RTS even during data overhearing without causing collisions at the source or destination [27,28]. That is, the neighbor can wake up before the NAV expires. In this paper, we utilize this feature to reduce the NAV.

**Figure 2:**
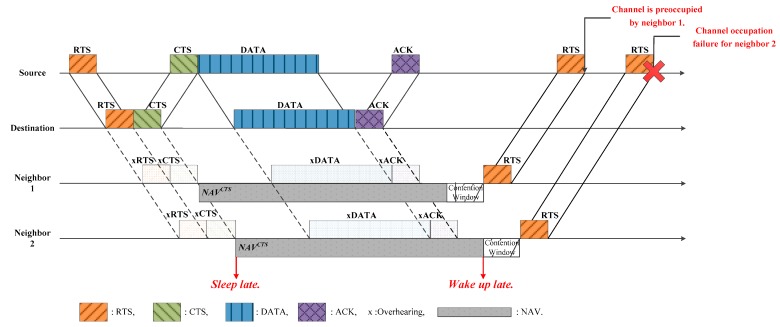
Illustration of the unfairness problem arising from inefficient NAV determination.

Another issue caused by the long propagation delay in underwater acoustic channels is the unfairness between nodes. Figure 2 shows a situation where the two neighbors 1 and 2 are waiting to access the channel. In case of a constant NAV, Neighbor 2, located farther away from the destination, overhears the CTS later than Neighbor 1, and accordingly, wakes up later. Thus, as long as Neighbor 1 has data to transmit, it occupies the channel, before Neighbor 2. This causes a severe unfairness problem.

## 3. Optimization of NAV

**Figure 3:**
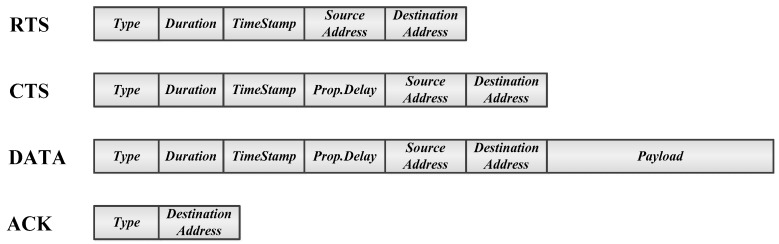
Packet structure of the proposed scheme.

Figure 3 shows the packet structure used in the proposed scheme. The *Duration* field, which is defined in typical CSMA/CA protocols, specifies the period from the instant the packet is transmitted to the instant the handshaking is expected to end. Thus, *Duration* has been used as the NAV in previous CSMA/CA protocols. The proposed scheme adds some message fields to help the neighbor obtain timing information and adjust the NAV accordingly, so that unnecessarily long silent periods or sleep are prevented. The additional message fields are *TimeStamp* and *Prop.Delay*. *TimeStamp* indicates the packet transmission time used by the associated receiver to calculate the propagation delay. *Prop.Delays* of CTS and DATA specify the propagation delays between the source and destination calculated by the destination and source, respectively. By overhearing the *TimeStamp* and *Prop.Delay*, a neighbor can acquire all propagation delays of τ(S,D), τ(D,N) and τ(S,N), where *S, D,* and *N* stand for the source, destination, and neighbor, respectively. 

**Figure 4:**
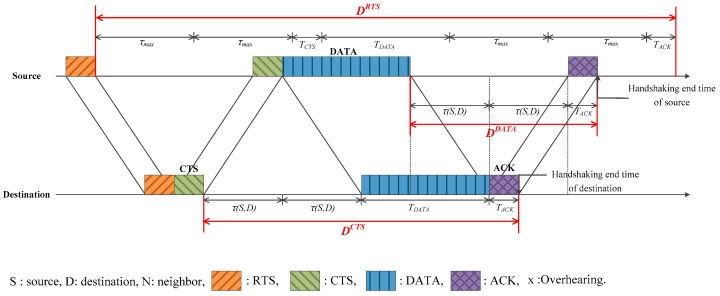
Duration of each packet.

The *Duration* is updated for every packet—RTS, CTS, and DATA—with the following values,

(3) $$D^{RTS}=4\tau_{max}+T_{CTS}+T_{DATA}+T_{ACK}$$

(4) $$D^{CTS}=2\tau (S,D)+T_{DATA}+T_{ACK}$$

(5) $$D^{DATA}=2\tau (S,D)+T_{ACK}$$

where $D^{X}$ denotes the *Duration* of packet *X*. As, at first, the source does not know τ(S,D), $\tau_{max}$ is used in $D^{RTS}$. Figure 4 shows the duration of each packet. Unlike previous CSMA/CA-based protocols, where *Duration* itself is used as the NAV, the proposed scheme eliminates the unnecessary parts in *Duration*, based on additional information such as the propagation delay and the node with whom the neighbor is trying to communicate after completion of the current handshaking. The detailed NAV-determination rule in each case is described in the following sections.

### 3.1. Trying to Communicate with Source

**Figure 5:**
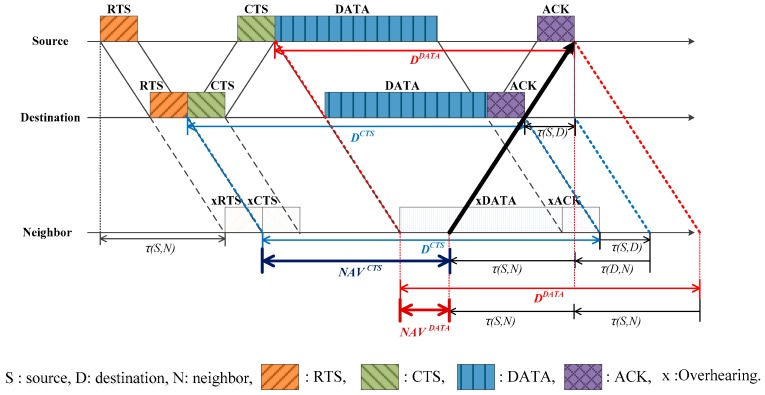
NAV determination when trying to communicate with source.

Figure 5 shows the case where a neighbor tries to occupy the channel after the current handshaking, in order to communicate with the source. After overhearing *DATA*, the neighbor reads $D^{DATA}$ and uses it to finalize the *NAV* as

(6) $$NAV^{DATA}=max\;[0,D^{DATA}-2\tau (S,N)]$$

where the subtraction of 2τ(S,N) corresponds to the propagation delays of DATA and the new RTS generated by the neighbor. Before determining $NAV^{DATA}$, the $NAV^{RTS}$ and $NAV^{CTS}$ are tentatively determined in the same way as $NAV^{DATA}$, as:

(7) $$NAV^{RTS}=max\;[0,D^{RTS}-2\tau (S,N)]$$

(8) $$NAV^{CTS}=max\;[0,D^{CTS}+\tau (S,D)-\tau (D,N)-\tau (S,N)]$$

In most cases, $NAV^{RTS}$ and $NAV^{CTS}$ are replaced by the latest $NAV^{DATA}$. However $NAV^{RTS}$ and $NAV^{CTS}$ must be preserved against DATA loss.

### 3.2. Trying to Communicate with the Destination

**Figure 6:**
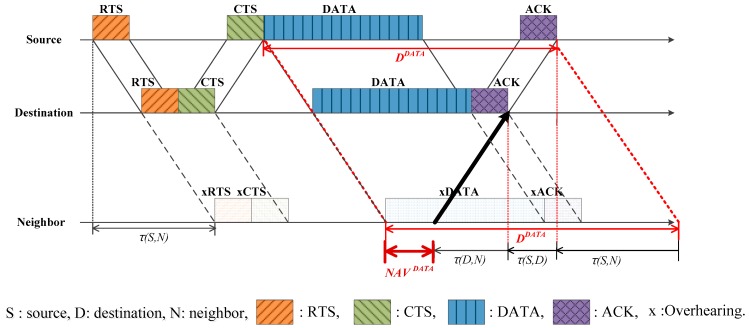
NAV determination when trying to communicate with destination.

Figure 6 shows the case where a neighbor tries to communicate with the destination, after the current handshaking. The new RTS departing from the neighbor (bold arrow) has to arrive right after the ACK transmission, at the destination. Thus, similar to the previous case, the final *NAV* is determined by

(9) $$NAV^{DATA}=max\;[0,D^{DATA}-\tau (S,N)-\tau (S,D)-\tau (D,N)]$$

In addition, the tentative NAVs are given by

(10) $$NAV^{RTS}=max\;[0,D^{RTS}-\tau (S,N)-\tau (S,D)-\tau (D,N)]$$

(11) $$NAV^{CTS}=max\;[0,D^{CTS}-2\tau (D,N)]$$

However, in Equation (10), since the neighbor does not know τ(S,D) and τ(D,N) at the moment of overhearing the RTS, $NAV^{RTS}$ is tentatively determined by

(12) $$NAV^{RTS}=max\;[0,D^{RTS}-\tau (S,N)]$$

### 3.3. Trying to Communicate with Another Neighbor

The propagation delays acquired during overhearing are the ones related to the source or destination only, and not the neighbors. Thus, in case a neighbor tries to communicate with another neighbor, the NAV is determined by the larger NAV out of those obtained in Section 3.1 and Section 3.2.

### 3.4. Extra NAV

**Figure 7:**
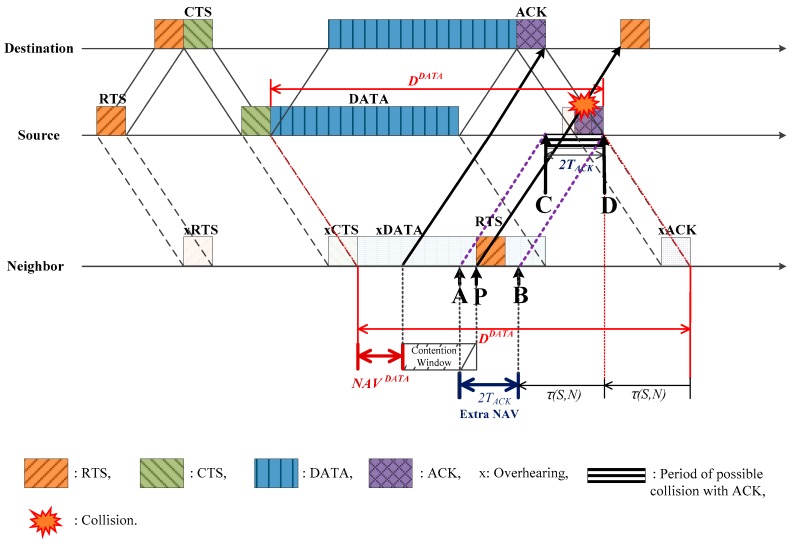
Extra NAV setting during destination–source–neighbor deployment.

In the case where a neighbor tries to communicate with the destination, and the source is located between the neighbor and the destination or the neighbor is located between the source and the destination, an additional silent period that we have named *extra NAV* is required to avoid possible collisions at the source. Figure 7 shows the case where the source is located between a neighbor and the destination, and the neighbor tries to send an RTS to initiate new communication with the destination. The RTS departing from the neighbor (point P), after the $NAV^{DATA}$ obtained by Equation (9) and a random back-off (denoted by the contention window in Figure 7), may collide at the source with the ACK that is supposed to be sent by the destination. In order to avoid such a collision, the neighbor should be silent during an additional period of $2T_{ACK}$, which is the *extra NAV*. In Figure 7, the point D denotes the time when the ACK reception is expected to be completed at the source and the point C is $2T_{ACK}$ ahead of D. Then, the period between points C and D is when the RTS arriving from the neighbor may collide with the ACK from the destination. Considering τ(S,N), the period between points A and B corresponds to the period between C and D, where

(13) B=D−τ(S,N)

(14) $$A=B-2T_{ACK}$$

In order to avoid collision, point P should be controlled such that it is not located within the period between A and B. That is, the neighbor needs that extra NAV. On the time line of the neighbor, point B is easily obtained as

(15) $$B=D^{DATA}-2\tau (S,N)$$

In a similar manner, the extra NAV for the case where a neighbor is located between the source and the destination is also obtained. Regarding the node arrangement, a neighbor is able to calculate the arrangement based on the propagation delays τ(S,D), τ(S,N), τ(D,N) which are calculated or obtained by referring to the message fields—*TimeStamp* and *Prop.Delay*. 

## 4. Performance Analysis

In this section, we evaluate the proposed NAV optimization scheme, comparing it to NAV setting methods described in [24,25] in terms of total NAV time, throughput, latency, and fairness. As mentioned earlier, both [24,25] are based on the CSMA/CA protocol with four-way handshaking mechanism and BEB (binary exponential back-off) algorithm [26], but differ in the method of NAV setting.

### 4.1. Simulation Conditions

An event-driven simulation was carried out in a network simulator developed in Matlab that has been used in our previous published works [22,23,29,30,31,32,33,34] and we considered Thorp’s underwater channel model [35,36]. We consider a 3-km × 3-km underwater plane at a certain depth, where 25 static nodes are randomly distributed. The main parameters used in the simulation are summarized in Table 1.

#### 4.1.1. NAV Time

**Figure 8:**
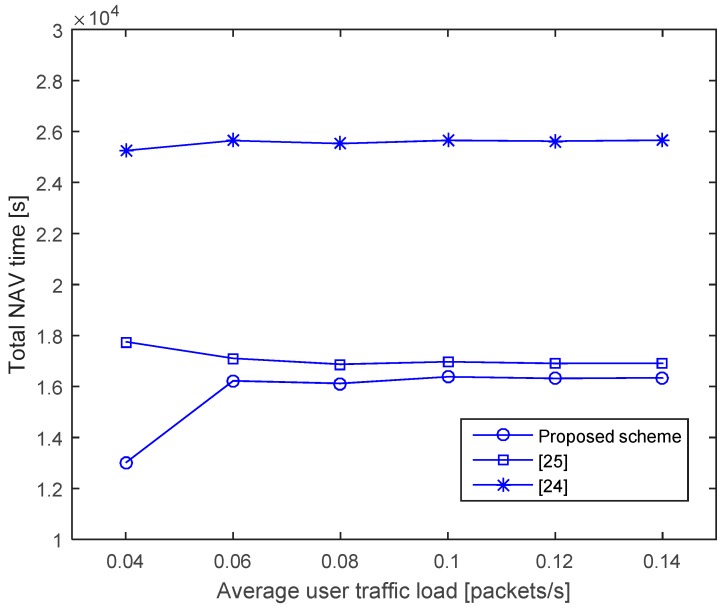
Total NAV time.

Figure 8 shows the total NAV, which is the sum of the NAVs of individual nodes with varying traffic loads. The user traffic is assumed to be generated by the Poisson arrival process at a rate between 0.04 and 0.14 packets per second. As shown in the figure, the proposed scheme and that of [25] have much smaller NAVs than the scheme in [24], as in [24], the NAV is extended for every packet overheard. In contrast, the proposed scheme and that of [25] selectively extend the NAV under specific conditions. However, the proposed scheme achieves better reduction of the NAV than in [25] by considering the internodal propagation delay. Not only does the proposed scheme achieve the lowest NAV times, it is also highly time efficient because unnecessary wait times are eliminated.

#### 4.1.2. Throughput

**Figure 9:**
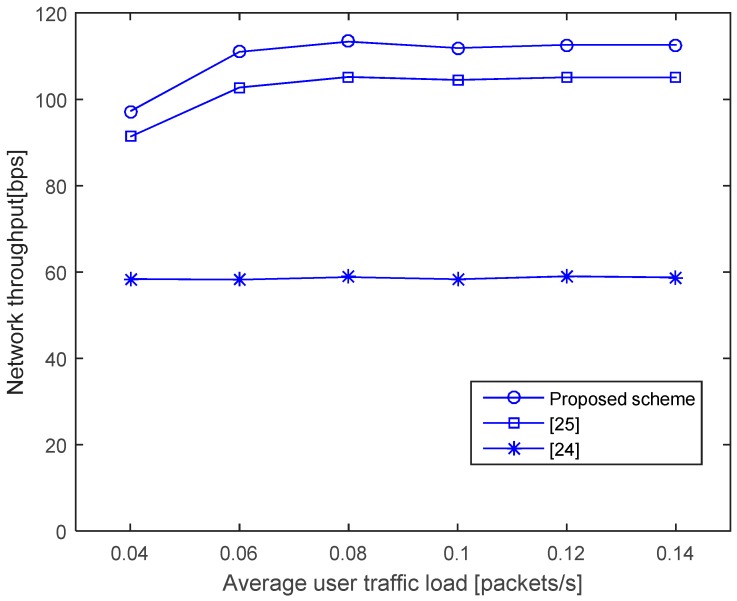
Network throughput.

Figure 9 shows the network throughput, which is defined as the aggregate number of bits successfully delivered to all destinations in the network, per second. It can be seen that the proposed scheme has the highest network throughput owing to the reduction of inefficient NAV time. In association with Figure 8, we can see that the lower network throughput is caused by a longer NAV time, which implies that an unnecessarily long NAV creates lost opportunities to access the channel even when it is available, degrading the throughput. 

#### 4.1.3. Latency

**Figure 10:**
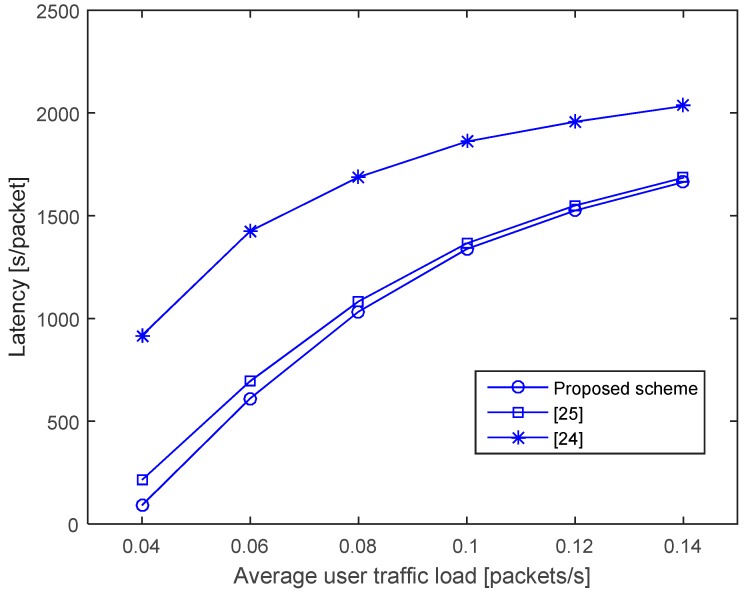
Latency.

Figure 10 shows the latency, which is defined as the average duration from the moment a packet is generated to the moment the packet is successfully delivered. For higher traffic loads, in general, the latency increases as collisions become more frequent, and the queueing delay until the channel is free also increases. Moreover, the latencies of the schemes with higher network throughputs (proposed scheme and [25]) are obviously lower because a higher network throughput also implies shorter queueing delays and fewer collisions. As expected, the proposed scheme achieves better latency as a consequence of optimizing the NAV.

#### 4.1.4. Fairness

There are two reasons for the unfairness existing between the nodes in CSMA/CA-based protocols. The first reason is that the number of neighbors varies considerably along the network because of the random deployment of the sensor nodes. Accordingly, a node with more neighbors might experience more packet collisions resulting in lower throughput. Such an uneven distribution of nodes causes a fairness issue in terms of throughput. The second reason is that the nodes farther away from the destination have a lower probability of occupying a channel than the ones closer to the destination, because the NAVs in the previous schemes are determined without considering the propagation delays between the nodes; thus, a distant node wakes up later and loses a chance to occupy the channel. The proposed scheme aims to eliminate the unfairness caused by the second reason, by determining the NAVs considering the propagation delays of the nodes, as discussed in Section 3.

**Figure 11:**
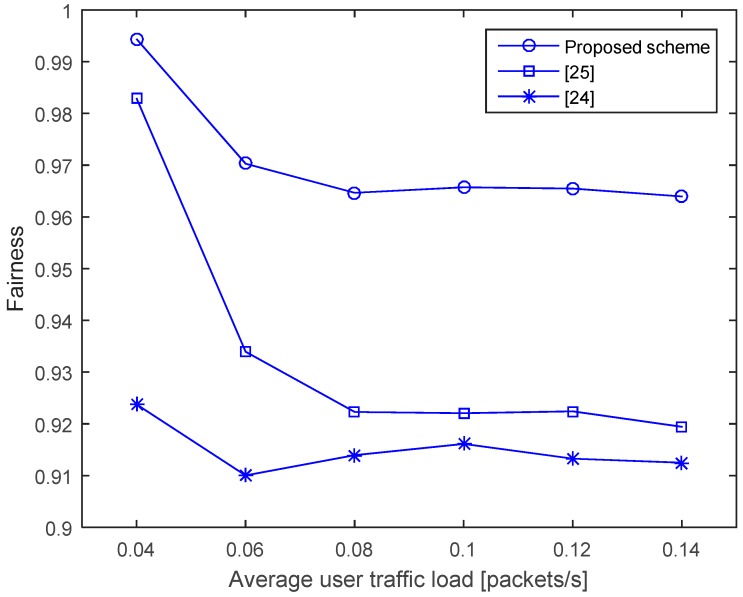
Fairness.

To measure the network’s fairness performance, the Jain’s fairness index [37] is used. For *n* nodes, the Jain’s fairness index is defined by

(16) $$J(x_{1},x_{2},\ldots ,x_{n})=\frac{{\left(\sum_{i=1}^{n}x_{i}\right)}^{2}}{n\times \sum_{i=1}^{n}{x_{i}}^{2}}$$

where *x_i_* is the throughput of node *i* (*i* = 1, 2, …, *n*). The index value ranges from $\frac{1}{n}$ (worst case, which occurs when only one node is able to deliver data while others deliver nothing) to 1 (best case, which occurs when all nodes deliver the same amount of data to the destination node). In Figure 11, it can be observed that the fairness decreases as the traffic increases, owing to the increase in the throughput differences among the nodes. As expected, the proposed scheme shows higher fairness values compared to the schemes in [24,25]. This is largely due to the fact that in the proposed scheme, the neighboring nodes have almost equal probability of channel occupation regardless of the node position—a consequence of considering the propagation delays of the nodes. Meanwhile, the fairness performance of [24] is the worst when compared to others, as [24] sets the NAV whenever it overhears a packet and a node that has many neighbors will have a relatively longer silent period, reducing its channel-occupation probability.

## 5. Conclusions

In this paper, we proposed a technique to optimize the NAVs used in underwater handshaking-based protocols such as the CSMA/CA protocol by eliminating the unnecessary long silent periods. To eliminate the unnecessary long silent periods, the proposed scheme supplies the neighbor nodes with the timing information about the propagation delays between a source and a destination, between a neighbor and a source, and between a neighbor and a destination, so that the neighbor can figure out the arrangement of the surrounding nodes and thus, find the precise wake-up time. Through computer simulations, it was shown that the elaborately determined NAV, which enables the precise wake-up time, improves the NAV time of the network. Similar improvements were also observed in the throughput and latency of the network, suggesting the importance of the NAV setting on these performance metrics. In addition, the use of optimized NAVs allows a distant neighbor node to have shorter silent periods and wake up earlier than the nearer ones to compensate for the longer distance from the sender/destination. This procedure effectively eliminates unfairness in channel occupation and was shown to improve the fairness of the network. Finally, compared to previous works on NAV settings for underwater handshaking-based protocols, it was shown that the proposed scheme outperforms them in every aspect.

## Figures and Tables

**Table 1:** System parameters.

Parameters	Value
Network dimensions	3 km × 3 km
Propagation speed	1500 m/s
Node transmission range	500 m
Data rate	1 kbps
Contention window size	15–1023 (binary exponential back-off)
